# New risk scoring system for predicting 3-month mortality after acute exacerbation of idiopathic pulmonary fibrosis

**DOI:** 10.1038/s41598-022-05138-6

**Published:** 2022-01-21

**Authors:** Susumu Sakamoto, Hiroshige Shimizu, Takuma Isshiki, Yasuhiko Nakamura, Yusuke Usui, Atsuko Kurosaki, Kazutoshi isobe, Yujiro Takai, Sakae Homma

**Affiliations:** 1 Division of Respiratory Medicine, Toho University Omori Medical Center, Ota-ku Omori nisi 6-11-1, Tokyo, 143-8541 Japan; 2 Department of Diagnostic Radiology, Fukujuji Hospital, Kiyose, Tokyo, Japan; 3 Department of Advanced and Integrated Interstitial Lung Diseases Research, School of Medicine, Toho University, Ota-ku, Tokyo, Japan

**Keywords:** Respiratory tract diseases, Risk factors

## Abstract

Acute exacerbation of idiopathic pulmonary fibrosis (AE-IPF) is often fatal. A straightforward staging system for AE-IPF would improve prognostication, guide patient management, and facilitate research. The aim of study is to develop a multidimensional prognostic AE-IPF staging system that uses commonly measured clinical variables. This retrospective study analyzed data from 353 consecutive patients with IPF admitted to our hospital during the period from January 2008 through January 2018. Multivariate analysis of information from a database of 103 recorded AE-IPF cases was used to identify factors associated with 3-month mortality. A clinical prediction model for AE-IPF was developed by using these retrospective data. Receiver operating characteristic (ROC) analysis was used to evaluate the diagnostic performance of this model. Logistic regression analysis showed that PaO_2_/FiO_2_ ratio, diffuse HRCT pattern, and serum C-reactive protein (CRP) were significantly associated with 3-month mortality; thus, PaO_2_/FiO_2_ ratio < 250 (P), CRP ≥ 5.5 (C), and diffuse HRCT pattern (radiological) (R) were included in the final model. A model using continuous predictors and a simple point-scoring system (PCR index) was developed. For the PCR index, the area under the ROC curve was 0.7686 (*P* < 0.0001). The sensitivity of the scoring system was 78.6% and specificity was 67.8%. The PCR index identified four severity grades (0, 1, 2, and 3), which were associated with a 3-month mortality of 7.7%, 29.4%, 54.8%, and 80%, respectively. The present PCR models using commonly measured clinical and radiologic variables predicted 3-month mortality in patients with AE-IPF.

## Introduction

Acute exacerbation of idiopathic pulmonary fibrosis (AE-IPF) does not respond to most conventional therapies and is thus frequently fatal^1–4^. The annual incidence of AE-IPF among patients with IPF is about 5% to 15%, and AE-IPF can occur at any time during the clinical course of IPF. AE-IPF can lead to death within a few weeks to a few months^3,4^; however, clinical course and survival vary considerably. Thus, predicting outcomes in patients with AE-IPF is an important challenge for clinicians.

Staging systems have proven useful for determining prognosis and guiding management decisions for lung diseases such as chronic obstructive pulmonary disease^5^, lung cancer^6^, and IPF^7,8^. Although some variables are known to be associated with AE-IPF mortality, no single variable accurately predicts outcomes^9–11^, and one clinical prediction model that combines variables has been proposed for AE-IPF^9^. A straightforward staging system for AE-IPF might improve prognostication, help guide management decisions, and allow for appropriate supportive care. Such a system could also improve future research on AE-IPF by identifying patients at high risk for clinically important outcomes.

We attempted to develop a multidimensional prognostic AE-IPF staging system that uses commonly measured clinical and radiological variables.

## Methods

### Study patients

**Figure 1:**
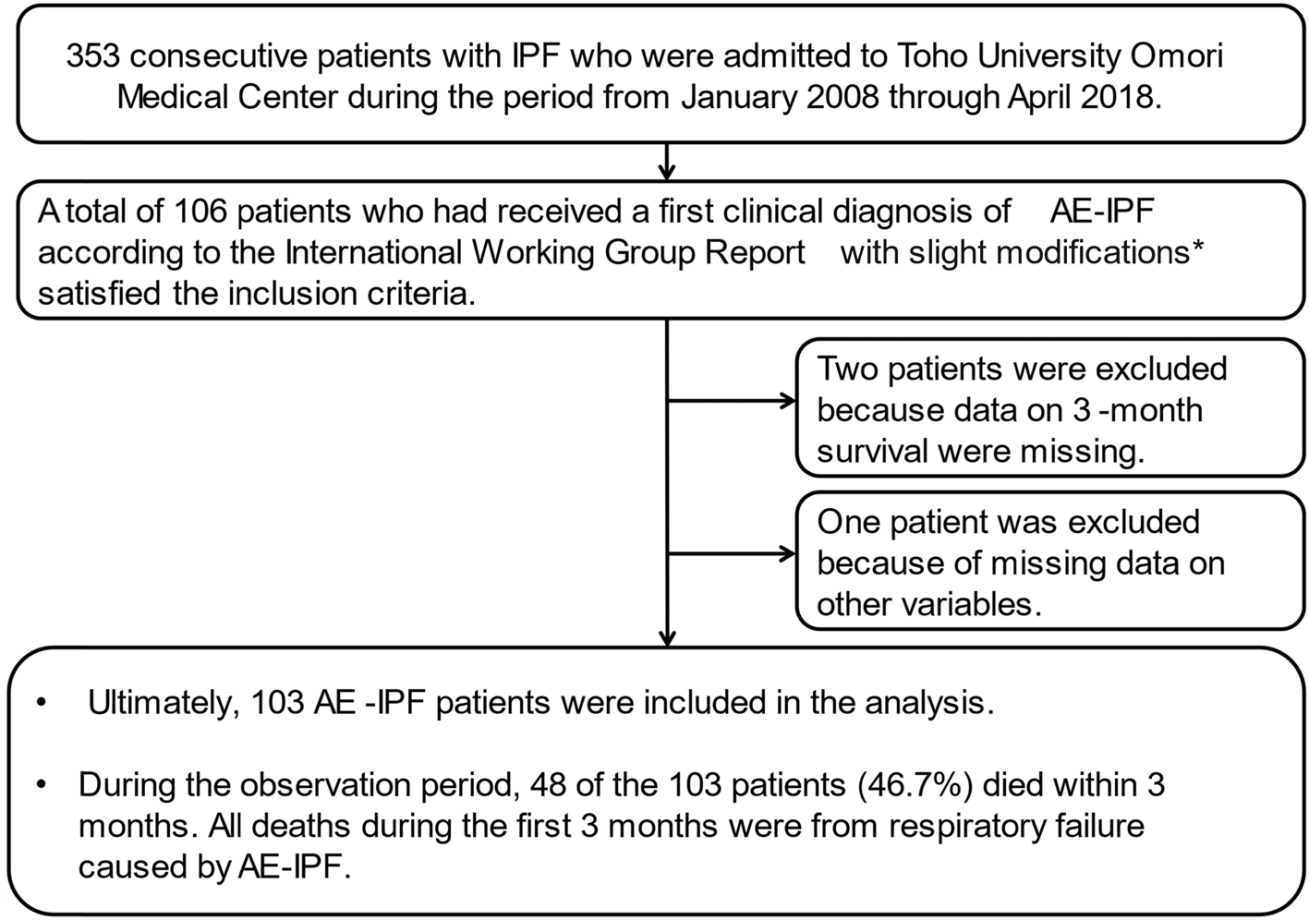
Flowchart of study. *AE-IPF was diagnosed when the following criteria were fulfilled (1) Previous or concurrent diagnosis of IPF, (2) Acute worsening or development of dyspnea typically < 1 month duration, (3) Computed tomography with new bilateral ground-glass opacity and/or consolidation superimposed on a background pattern consistent with UIP pattern, (4) deterioration not fully explained by cardiac failure, fluid overload (5) no evidence of pulmonary infection on bronchoalveolar lavage, endotracheal aspiration, or sputum culture and negative results on blood tests for other potentially infectious pathogens).

This retrospective study investigated data from 353 consecutive patients with IPF who were admitted to Toho University Omori Medical Center during the period from January 2008 through April 2018. A total of 106 patients who had received a first clinical diagnosis of AE-IPF satisfied the inclusion criteria. Two patients were excluded because data on 3-month survival were missing. One patient was excluded because of missing data on other variables. Ultimately, 103 AE-IPF patients were included in the analysis. The median duration of observation from the first visit to our center was 13 months (range 1–137 months). During the observation period, 48 of the 103 patients (46.7%) died within 3 months. All deaths during the first 3 months were from respiratory failure caused by AE-IPF (Fig. 1).

### Data collection

Clinical data were collected to determine the characteristics of underlying IPF and IPF treatment before AE. We also collected information on respiratory function during the 6-month period before AE. The analyzed covariates included PaO_2_/FiO_2_ ratio and serological tests, namely, C-reactive protein (CRP), lactate dehydrogenase (LDH), Krebs von den Lungen-6 (KL-6), and surfactant protein D (SP-D).

### Diagnosis of IPF and AE-IPF

IPF was diagnosed in accordance with the 2018 international IPF guideline^10^. All patients had a pathological usual interstitial pneumonia (UIP) or UIP pattern on chest high-resolution computed tomography (HRCT), which was confirmed by histological examination of lung biopsy specimens, and/or satisfied the clinical diagnostic criteria for IPF. CT images and clinical records of patients with suspected IPF were subsequently reviewed by 1 thoracic radiologist (A.K.) and 5 pulmonologists (S.S., H.S., T.I., Y.N., and H.S.), who reached a consensus diagnosis regarding clinical IPF. IPF severity before AE was assessed with the Gender-Age-Physiology (GAP) index, which is calculated by using data on gender, age, forced vital capacity (FVC)% predicted, and diffusion capacity (DLco)% predicted. Disease severity was classified as stage I–III, as previously described. Disease severity, pulmonary function, IPF treatment, and diagnostic findings on HRCT were evaluated while IPF was chronic and stable, i.e., before AE-IPF onset.

AE-IPF was defined in accordance with the International Working Group Report^1^, with slight modifications, and was diagnosed when the following criteria were fulfilled (1) Previous or concurrent diagnosis of IPF, (2) Acute worsening or development of dyspnea (duration, typically < 1 month), (3) Computed tomography findings showing a new bilateral ground-glass opacity and/or consolidation superimposed on a background pattern consistent with UIP pattern, (4) deterioration not fully explained by cardiac failure or fluid overload, and (5) no evidence of pulmonary infection on bronchoalveolar lavage, endotracheal aspiration, or sputum culture and negative results on blood tests for other potentially infectious pathogens (e.g., *Pneumocystis jirovecii*, cytomegalovirus).

### Evaluation of HRCT images of AE-IPF

HRCT images of all the present patients were reviewed by 3 pulmonologists (H.S., T. I., S.S.) and 1 chest radiologist (A.K.) who were blinded to the clinical characteristics of the patients. Using the classification of Akira et al.^11^, we classified the CT pattern at AE-IPF onset as diffuse or non-diffuse. HRCT images at AE-IPF onset were reviewed by evaluating 3 images, namely, those at the level of the aortic arch, carina, and 1 cm above the diaphragm. In the patients studied, new pulmonary opacities at acute exacerbation exhibited different degrees of increased opacity (mixed areas of ground-glass attenuation and consolidation). In semiquantitative analysis, ground-glass opacity and consolidation were grouped together as alveolar opacity. Each lung lobe was evaluated to determine the percentage of newly developed alveolar opacity in relation to the extent and distribution of involvement, except for honeycomb and cystic areas. The percentage for each lobe was defined as the average of the values from all investigators. A diffuse HRCT pattern was defined as an extent of involvement of newly developed alveolar opacity greater than 50%.

### Primary outcome

The primary outcome was 3-month mortality. Vital status and date of death were verified by using the information in the database. Using a database of 103 cases of AE-IPF recorded at our hospital, we used multivariate analysis to identify factors associated with 3-month mortality. We selected clinical variables reported to be associated with AE-IPF outcome^9,11,12,14–18^ at the time of AE-IPF diagnosis because our aim was to establish a scoring system that could be used even for patients with no previous data. The results of multivariate analysis of factors significantly associated with 3-month mortality were used to identify variables that could be used as candidates in the model. An AE-IPF clinical prediction model was developed by using retrospective data from our cohort, and logistic regression was used to evaluate the model. We retrospectively screened potential predictors of mortality in patients with AE-IPF, which yielded a model comprising 3 predictors. On the basis of these predictors, we then developed a simple point-score model and staging system (PCR index and staging system).

### Statistical analysis

Statistical analysis was performed with IBM SPSS Statistics software (version 26). Patients with missing data were excluded from the analysis. Multivariate logistic regression analysis was performed with clinical variables at AE-IPF onset, namely, age, serum KL-6, SP-D, CRP, and LDH concentrations, PaO_2_/FiO_2_ ratio, HRCT pattern (diffuse vs non-diffuse), and antifibrotic agent use as explanatory variables, followed by stepwise variable selection (backward elimination with a threshold of *P* = 0.05). The values for selected variables in the multivariate analysis were categorized by using cut-off values determined by receiver operating characteristic (ROC) curve analysis. Then, multivariate logistic regression analysis with the categorized variable was again performed to validate its significance. Each selected variable was assigned an integral weight proportional to its partial regression coefficient. For all patients, the total score was defined as the sum of the values for the selected variable. The association between total score and 3-month mortality for AE-IPF was investigated by using ROC curves, and the predicted 3-month mortality cut-off value was calculated. To calibrate the PCR model, we used the Hosmer–Lemeshow test to evaluate agreement between the probability predicted by PCR score and observed probability (calibration plot). In addition, we performed internal validation by using the bootstrap method. Furthermore, to confirm consistency with the lifetime distribution of patients, we constructed Kaplan–Meier survival curves for each PCR index and used the log-rank test for trend to test the correspondence between the temporal trend of the curves and the PCR index. A *P* value of < 0.05 was considered to indicate statistical significance.

### Ethical approval

This retrospective study was approved by the Institutional Review Board of Toho University Omori Medical Center on June 18, 2019 (project approval number, M18259). Considering the nature of the retrospective study, the informed consent was waived by the IRB because of the anonymized patient data. The study protocol was performed in accordance with the relevant guidelines.

## Results

### Patient characteristics

The characteristics of the 103 patients with AE-IPF are shown in Table 1. Among the 88 men and 15 women (mean age, 73.7 years; range, 55–89 years) there were 3 current smokers, 81 former smokers, and 19 never-smokers. Mean (SD) number of pack-years was 57.9 (31.2), and 94 cases were associated with a UIP pattern and 9 cases were associated with a probable UIP pattern. All cases of probable UIP pattern were diagnosed as UIP by surgical lung biopsy or autopsy. Ten patients (9.7%) had a pathological diagnosis of UIP, as determined by analysis of a surgical lung biopsy specimen obtained 0–36 months before AE-IPF onset (n = 7) or by autopsy (n = 3). Table 1 also shows data for laboratory variables, interstitial lung disease markers (such as KL-6 and SP-D), HRCT pattern of AE-IPF, and PaO_2_/FiO_2_ ratio at AE-IPF onset. The detailed clinical characteristics of the patients before AE onset (0–6 months) are also shown in Table 1. PaO_2_/FiO_2_ ratio at AE-IPF onset was significantly but weakly negatively correlated with GAP stage (r =  − 0.2937, *P* < 0.05) and JRS classification of IPF disease severity (r =  − 0.3986, *P* < 0.01). All patients were treated with high-dose corticosteroid pulse therapy (methylprednisolone 1000 mg/day for 3 days). Corticosteroid dose was tapered after pulse therapy (0.5–1.0 mg/kg/day). Some patients were treated with combination treatment: cyclosporin A (CsA) 2.5 mg/kg/day was administered orally, sivelestat 4.8 mg/kg/day was administered intravenously for the first 14 days, and recombinant human thrombomodulin (rhTM) 0.06 mg/kg/day was administered intravenously for the first 6 days.

**Table 1:** Characteristics of 103 patients with AE-IPF.

	Overall (n = 103)	Survivors (n = 55)	Non-survivors (n = 48)	*P* value
Male sex n (%)	88 (85.4%)	45(81.8%)	43(89.6%)	0.4
Age median (range)	73.7 ± 6.9	73.4 ± 7.2	74.1 ± 6.6	0.76
*Smoking history*				
Never/former/current	19/81/3	10/44/1	9/37/2	0.98
Pack-years (mean ± SD)	57.9 ± 31.2	58.3 ± 29.3	56.4 ± 34.3	0.56
%FVC	71.0 ± 71.8	72.9 ± 21.0	68.6 ± 19.1	0.44
%DLco	35.5 ± 30.4	37.2 ± 29.6	33.6 ± 31.4	0.57
GAP score	2.7 ± 2.2	2.8 ± 2.3	2.6 ± 2.2	0.68
*Laboratory and radiological findings at the onset of AE-IPF*				
PaO_2_/FiO_2_ ratio	252.2 ± 85.6	283.3 ± 79.3	217.4 ± 79.3	< 0.001*
KL-6 (U/ml)	1535.3 ± 1856.8	1291.4 ± 875.2	1748.2 ± 2397.2	0.58
SP-D (ng/ml)	438.6 ± 356.6	395.9 ± 321.9	487.6 ± 390.3	0.22
SP-A (ng/ml)	103.0 ± 58.5	101.9 ± 60.6	104.4 ± 56.5	0.98
LDH (U/l)	373.9 ± 186.1	329.1 ± 68.7	424.3 ± 253.3	0.007*
CRP (mg/dl)	8.0 ± 6.4	6.2 ± 5.5	10.0 ± 6.9	0.002*
Diffuse HRCT pattern, n (%)	73 (70.9)	30 (54.5)	43 (89.6)	< 0.001*

%FVC, Forced vital capacity, % of predicted value; %DLco, Carbon monoxide diffusing capacity -% of predicted value; GAP score. Gender-Age-Physiology score; KL-6, Krebs von der Lungen-6; SP-D, surfactant protein D; SP-A, surfactant protein A; LDH, lactate dehydrogenase; CRP, C-reactive protein; HRCT, high-resolution computed tomography; CS, corticosteroid, PMX-DHP, Polymyxin B-immobilized fiber column-direct hemoperfusion; rhTM, recombinant human thrombomodulin.

*P < 0.05.

### Logistic regression analysis for 3-month mortality

In multivariate logistic regression analysis with selected explanatory variables, 3-month mortality was significantly associated with PaO_2_/FiO_2_ ratio (OR 0.9890, 95% CI 0.9816–0.9965, *P* = 0.0041), diffuse HRCT pattern (OR 3.8373, 95% CI 1.1395–12.9224, *P* = 0.0299), serum KL-6 concentration (OR 0.9993, 95% CI 0.9986–0.9999, *P* = 0.0353) and serum CRP concentration (OR 1.0901, 95% CI 1.0082–1.1786, *P* = 0.0303) (Table 2). Age (OR 0.9635, 95% CI 0.8914–1.0415, *P* = 0.3491), antifibrotic agent use (OR 0.1669, 95% CI 0.0755–1.1817, *P* = 0.0851), serum LDH concentration (OR 1.0045, 95% CI 0.9977–1.0114, *P* = 0.1972), and serum SP-D concentration (OR 1.0009, 95% CI 0.9993–1.0026, *P* = 0.2711) were not associated with 3-month mortality. Neither the severity of IPF nor pulmonary function test results before AE-IPF were significantly associated with 3-month mortality. Furthermore, no AE-IPF treatment was significantly associated with 3-month mortality. Thus, these factors were not used as candidate variables in multivariate analysis.

**Table 2:** Results of multivariate logistic regression analysis with selected variables as explanatory variables (upper) and after stepwise variable selection (lower).

Variables	Partial regression coefficient	95% CI of partial regression coefficient	Odds ratio	95% CI of Odds ratio	*P* value
Age	− 0.0372	− 0.1150 to 0.0407	0.9635	0.8914 to 1.0415	0.3491
Diffuse HRCT pattern	1.3448	0.1306 to 2.5590	3.8373	1.1395 to 12.9224	0.0299*
KL-6	− 0.0007	− 0.0014 to 0.0001	0.9993	0.9986 to 0.9999	0.0353*
SP-D	0.0009	− 0.0007 to 0.0026	1.0009	0.9993 to 1.0026	0.2711
LDH	0.0045	− 0.0023 to 0.0114	1.0045	0.9977 to 1.0114	0.1972
CRP	0.0863	0.0082 to 0.1643	1.0901	1.0082 to 1.1786	0.0303*
PaO_2_/FiO_2_ ratio	− 0.0110	− 0.0186 to 0.0035	0.9890	0.9816 to 0.9965	0.0041*
Antifibrotic agents use	− 1.2087	− 2.5842	0.1669	0.0755 to 1.1817	0.0851

Variables	Partial regression coefficient	95% CI of partial regression coefficient	Odds ratio	95% CI of odds ratio	*P* value
*Stepwise variable selection*					
Diffuse HRCT pattern	1.3386	0.2430 to 2.4342	3.8137	1.2751 to 11.4065	0.0166*
CRP	0.0943	0.0217 to 0.1669	1.0989	1.0219 to 1.1817	0.0109*
PaO_2_/FiO_2_ ratio	− 0.0082	− 0.0142 to 0.0022	0.9919	0.9859 to 0.9978	0.0074**

CI, confidence interval; HRCT, high-resolution computed tomography; KL-6, Krebs von der Lungen-6; SP-D, surfactant protein D; SP-A, surfactant protein A; LDH, lactate dehydrogenase; CRP, C-reactive protein.

*P < 0.05; **P < 0.01.

In multivariate logistic regression analysis with selected explanatory variables, followed by stepwise variable selection, 3-month mortality was significantly associated with PaO_2_/FiO_2_ ratio (OR 0.9919, 95% CI 0.9859–0.9978, *P* = 0.0074), diffuse HRCT pattern (OR 3.8137, 95% CI 1.2751–11.4065, *P* = 0.0166), and serum CRP concentration (OR 1.0989, 95% CI 1.0219–1.1817, *P* = 0.0109) (Table 2). The c-index was 0.8027 (95% CI 0.7167–0.8886, *P* < 0.0001). In addition, 1-month mortality was significantly associated with PaO_2_/FiO_2_ ratio (OR 0.9910, 95% CI 1.0179–0.9973, *P* = 0.0053) and serum CRP concentration (OR 1.0965, 95% CI 1.0179–1.1812, *P* = 0.0152) in multivariate analysis.

### New risk scoring system for predicting 3-month mortality after AE-IPF

**Figure 2:**
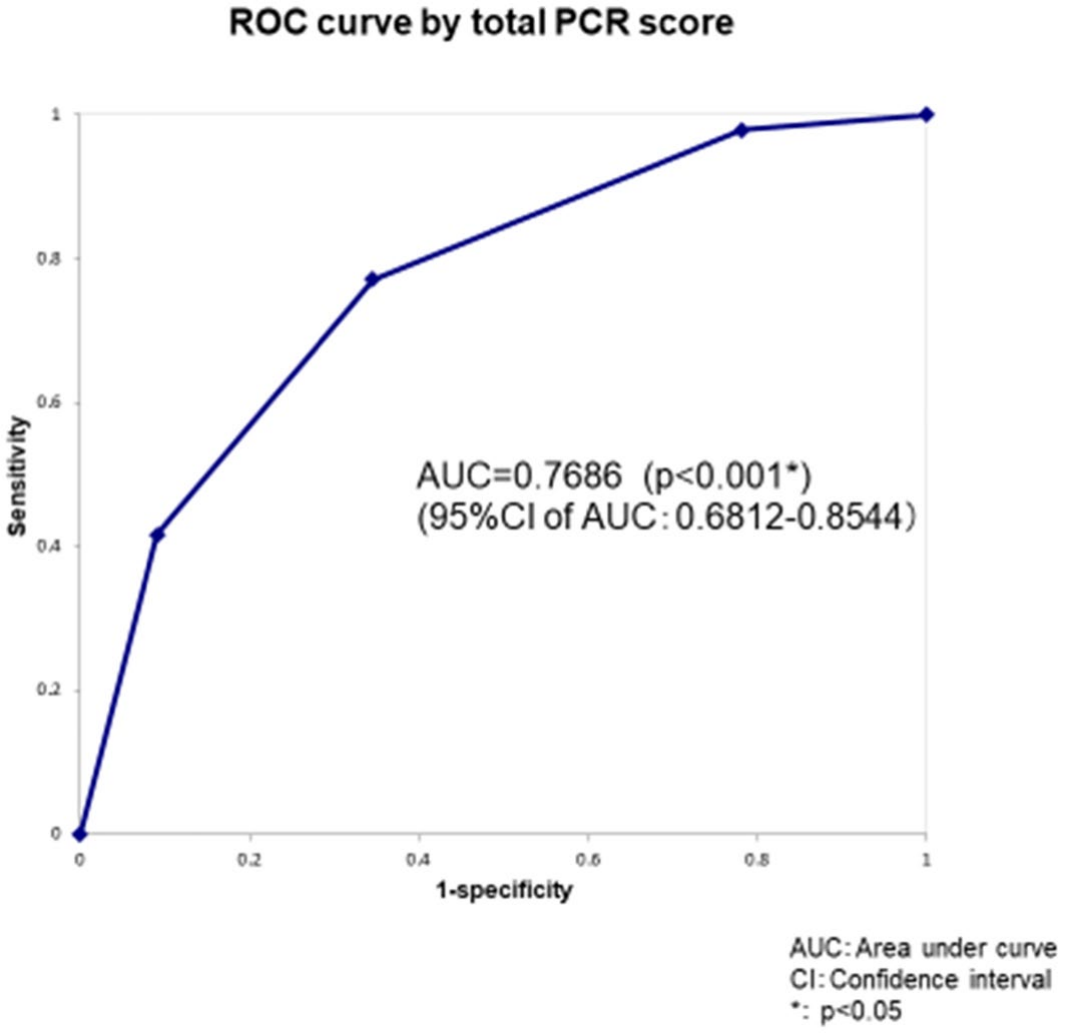
Receiver operating characteristic (ROC) analysis and diagnostic performance of PCR score for predicting 3-month mortality. The area under the ROC curve showed that PCR score successfully predicted survivors and nonsurvivors (AUC (c-index) = 0.7686; 95% CI 0.6812–0.8544; *P* < 0.001).

**Figure 3:**
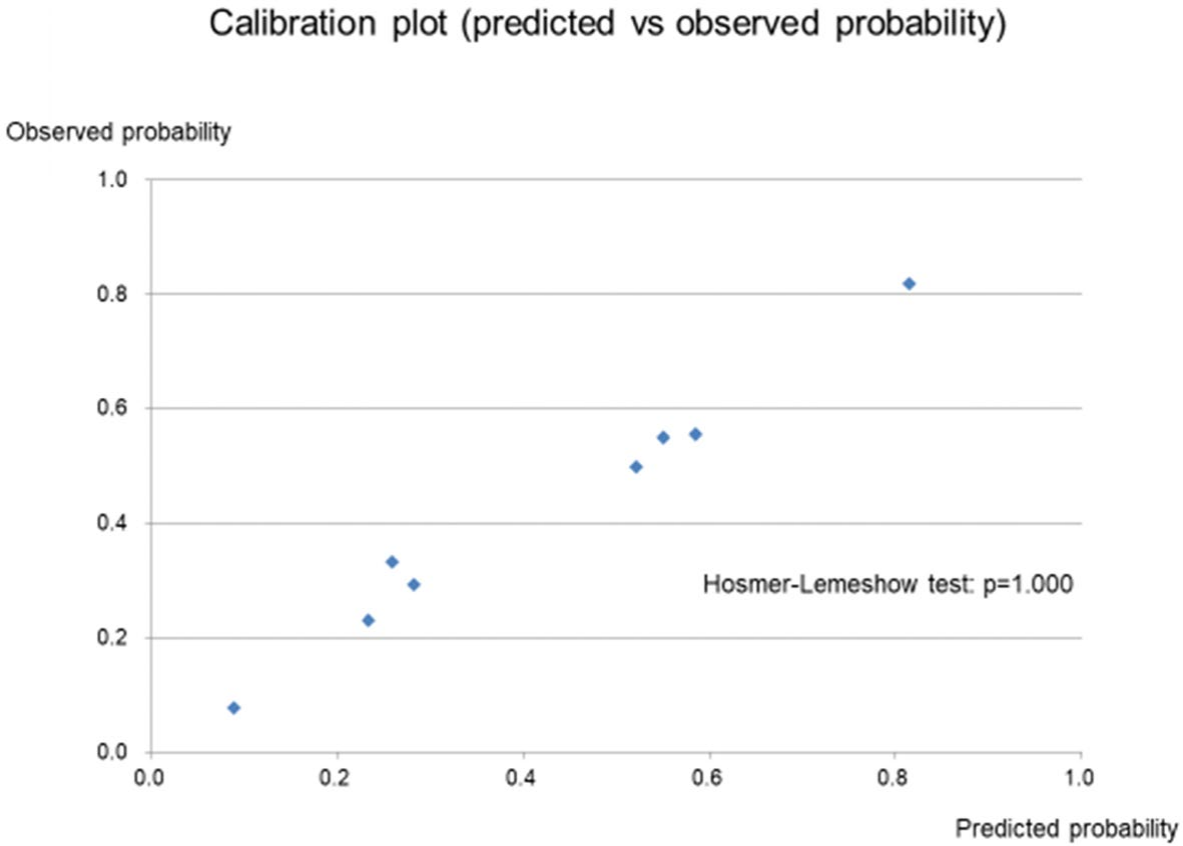
Calibration plot between the probability predicted by PCR score and observed probability. The Hosmer–Lemeshow test confirmed that the observed and predicted probabilities were in excellent agreement.

**Figure 4:**
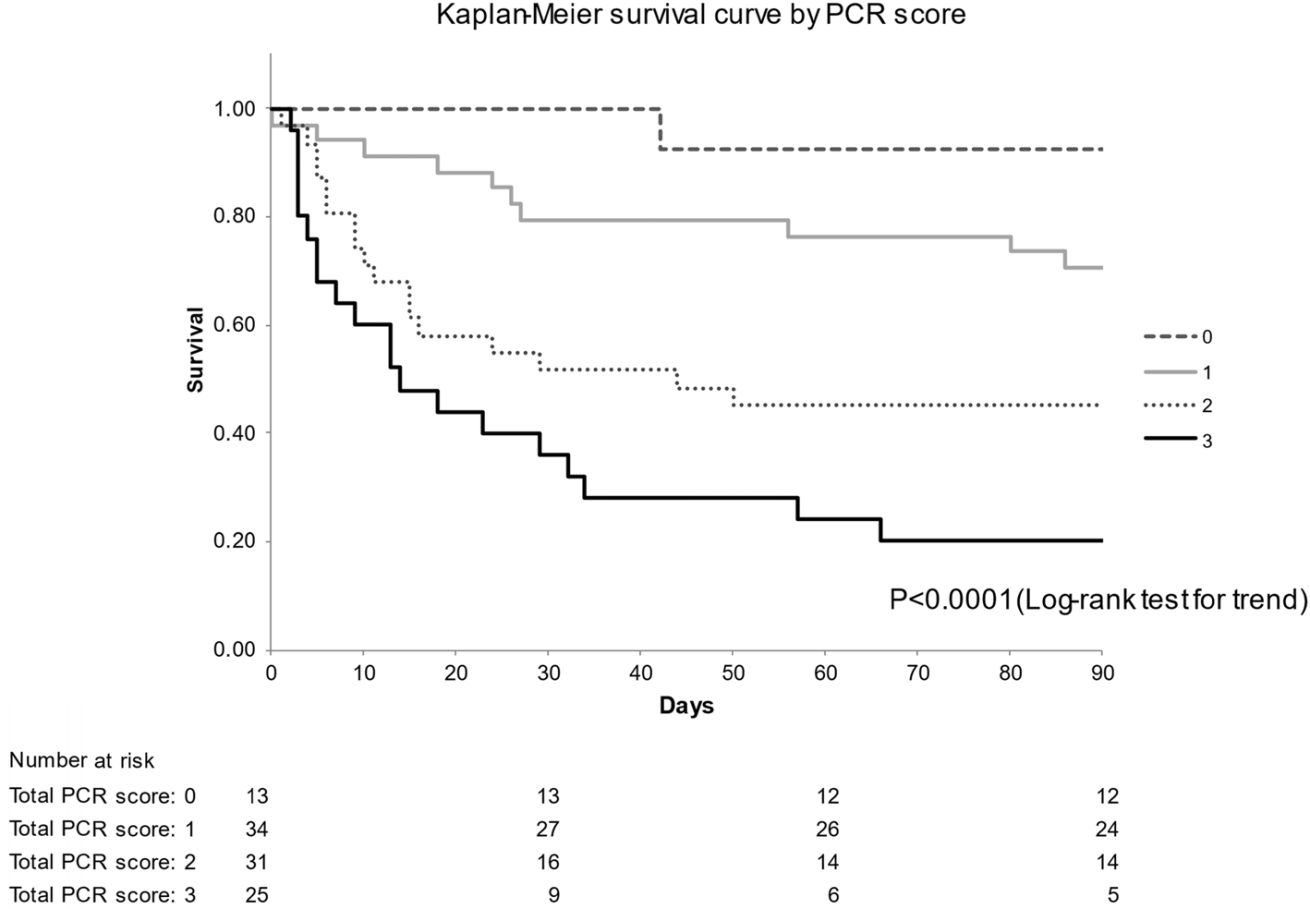
Kaplan–Meier survival curves for 3-month survival after onset of acute exacerbation of idiopathic pulmonary fibrosis, by PCR score. Three-month mortality was 7.7% for a score of 0, 29.4% for a score of 1, 54.8% for a score of 3, and 80% for a score of 4. The log-rank test for trend revealed that survival curves were consistent with PCR scores (*P* < 0.0001).

To develop the grading system, cut-off values for variables were determined by ROC curve analysis. The cut-off value was 250 for PaO_2_/FiO_2_ ratio and 5.5 for CRP concentration. The variables identified as significant risk factors for 3-month mortality were a PaO_2_/FiO_2_ ratio of < 250 (OR 3.1632, 95% CI 1.2497–8.0065, *P* = 0.0151), diffuse HRCT pattern (OR 4.1872, 95% CI 1.4445–12.1378, *P* = 0.0084), and serum CRP concentration ≥ 5.5 (OR 2.7347, 95% CI 1.1045–6.7710, *P* = 0.0296), all with integral weights of 1 (Table 3). The c-index was 0.7739 (95% CI 0.6855–0.8623, *P* < 0.0001). Points were assigned to variable categories to create a point-score model (PCR index), as shown in Table 4. The total score (PCR score) was calculated as [1 × PaO2/FiO2 ratio (< 250): (P)] + [1 × CRP (≥ 5.5 mg/dl): (C)] + [1 × HRCT (radiological) findings (diffuse HRCT pattern): (R)]. The score ranged from 0 to 3. The diagnostic performance of the PCR score for predicting 3-month mortality was examined by ROC analysis, and the area under the ROC curve (AUC) was 0.7686 (95% CI 0.6812–0.8544, *P* < 0.0001). The optimal cut-off value for PCR score was 2, as indicated by the Youden index, and sensitivity was 0.771 and specificity was 0.655 for predicting cause-specific survival (Fig. 2). To calibrate the PCR model, we evaluated agreement between the predicted probability by PCR score and observed probability. Figure 3 shows the calibration plot. The observed and predicted probabilities were in excellent agreement in the Hosmer–Lemeshow test (*P* = 1.000). Ultimately, a grading system was created by grouping point scores into 4 groups. Three-month mortality was 7.7% for a score of 0, 29.4% for a score of 1, 54.8% for a score of 3, and 80.0% for a score of 4 (Fig. 4). The log-rank test for trend revealed that survival curves were consistent with PCR scores (*P* < 0.0001). In addition, we confirmed that values for Kaplan–Meier analysis of the 3-month mortality rate for each PCR score and the estimated 3-month mortality rate calculated by the bootstrap method exhibited similar trends (Table 4).

**Table 3:** Results of multivariate logistic regression analysis with categorized selected variables.

Variables	Partial regression coefficient	95% CI of partial regression coefficient	Odds ratio	95% CI of odds ratio	*P* value	Integral weight
Diffuse HRCT pattern	1.4320	0.3677–2.4963	4.1872	1.4445–12.1378	0.0084*	1
High CRP (≥ 5.5)	1.0060	0.0994–1.9126	2.7347	1.1045–6.7710	0.0296*	1
Low PaO_2_/FiO_2_ ratio (< 250)	1.1516	0.2229–2.0802	3.1632	1.2497–8.0065	0.0151*	1

CI, confidence interval; HRCT, high-resolution computed tomography; CRP, C-reactive protein.

*P < 0.05.

**Table 4:** PCR index and grading system.

Predictor	Value	Score			
PaO_2_/FiO_2_ ratio (P)	< 250	1			
	$\geqq$ 250	0			
CRP (C)	$\geqq$ 5.5	1			
	< 5.5	0			
HRCT pattern (R)	Diffuse	1			
	Non-diffuse	0			

Total score of PCR index	Measured value (n = 103)		3-Month mortality estimated by bootstrap method	95% CI	
	No. of patients	3-month mortality		Lower	Upper
*Total possible points 3*					
0	n = 13	0.077 (7.7%)	0.083 (8.3%)	0.072	0.081
1	n = 34	0.294 (29.4%)	0.291 (29.1%)	0.291	0.300
2	n = 31	0.548 (54.8%)	0.554 (55.4%)	0.564	0.576
3	n = 25	0.800 (80.0%)	0.803 (80.3%)	0.798	0.808

CI, confidence interval; HRCT, high-resolution computed tomography; CRP, C-reactive protein.

## Discussion

We developed a multidimensional PCR index and grading system and a PCR calculator that use commonly measured clinical, laboratory, physiological, and radiological variables to predict AE-IPF mortality. The index and grading system are based on an easily calculated point score that has discriminatory power similar to that of its more complex continuous model, and to that of prognostic models used widely for other diseases. The PCR index and staging system can be used as a simple method of risk “screening” for patients with AE-IPF, and the PCR calculator is suitable for selected patients for whom precise risk assessment could alter management.

Putative prognostic factors for AE-IPF include CRP, LDH, HRCT pattern (score), PaO_2_/FiO_2_ ratio, and KL-6^9,11–16^. Kishaba et al. proposed a system for staging AE-IPF^9^. Their multivariate analysis identified 4 parameters: serum LDH, KL-6, PaO_2_/FiO_2_ ratio, and the overall extent of abnormal findings on chest HRCT (CT score). Patients were then classified as having extensive or limited disease on the basis of the 4 composite parameters. Patients with extensive disease were more likely to require mechanical ventilation and intensive therapy than were those with limited disease. In addition, 3-month mortality was higher in patients with extensive disease. The diagnostic performance in predicting 3-month mortality was examined by ROC analysis, and the area under the ROC curve significantly differed between survivors and nonsurvivors (AUC = 0.76; 95% CI 0.7–0.82). Although this staging system accurately reflected outcomes, it includes only 2 stages and is somewhat crude. Furthermore, this grading requires complicated CT score calculations. In contrast, the PCR score does not require complicated CT score calculation, as, at diagnosis, only 3 variables have discriminatory power (AUC = 0.768), which similar to that of Kishaba’s continuous model. Moreover, PCR score can be classified into 4 stages, which enables more detailed prediction of outcome.

We believe that the PCR score is complimentary and has important implications for clinical practice and research. Specifically, we believe that these tools could provide clinicians and patients with a framework for discussing prognosis with a tool to investigate stage-specific management options and researchers with the means to identify at-risk study populations, which could maximize the efficiency and statistical power of clinical trials.

Akira et al.^11^ classified the pattern of HRCT pulmonary opacification, such as ground-glass attenuation and consolidation, as peripheral, multifocal, and diffuse at AE-IPF onset. The diffuse HRCT pattern was associated with a poor prognosis. Because this classification is relatively straightforward for pulmonary physicians, we used it to classify HRCT findings and confirmed that a diffuse HRCT pattern was associated with poor prognosis.

A previous study evaluated the pattern, distribution, and extent of HRCT findings at patient presentation and calculated the HRCT score at AE-IPF by examining areas of normal attenuation and the extent of abnormalities, including areas of ground-glass attenuation or consolidation, with or without traction bronchiectasis or bronchiolectasis, and areas of honeycombing. Univariate analysis identified serum KL-6 level, PaCO_2_, and HRCT score as significant predictors; however, multivariate analysis showed that only HRCT score significantly independently predicted outcome. The authors concluded that HRCT score at AE-IPF onset was independently related to outcome in patients with AE-IPF. The HRCT scoring system was adapted from the classification of Ichikado et al., which was previously shown to be an independent prognostic factor for patients with acute respiratory distress syndrome, sepsis, and acute interstitial pneumonia^17,18^. Although this scoring system is excellent for predicting prognosis, it might be too complicated for clinical use by pulmonary physicians.

In the present analysis, a PaO_2_/FiO_2_ ratio of < 250, CRP of ≥ 5.5, and a diffuse HRCT pattern at AE onset were identified as prognostic factors and scored. A cut-off of 2 points was used to determine risk of 3-month mortality after AE-IPF. AE-IPF is thought to be attributable to multiple interrelated risk factors, and the present risk scoring system is likely to be useful clinically.

PCR models have important advantages over previously developed AE-IPF prediction models^9^. First, the present predictors are simple to ascertain and are multidimensional, as they incorporate clinical, physiological, and radiological variables. Second, extension of the PCR index into a grading system provides a framework for grade-specific treatment and research recommendations.

Future studies should examine the effect of PCR models on disease management. Expanded models that incorporate more-complex baseline variables (e.g., severity of IPF before AE and biomarkers) and longitudinal measurements (e.g., change in FVC or DLco before AE) should be compared with the present simple model to determine their additional prognostic value, if any, before incorporation into clinical practice.

## Limitations

The present cohort was analyzed retrospectively, and the small sample size may affect data quality. In addition, our cohort was drawn from an academic center; thus, because of referral bias, the included patients might differ from the general population of patients with AE-IPF. In addition, the present scoring system was not validated with another cohort. Furthermore, to make the model more universally applicable, we excluded variables that are not always available to clinicians at initial or subsequent patient visits, such as serial change in lung function. Finally, although we took pains to exclude patients with clinical pulmonary infection or heart failure, the difficulty of diagnosing AE-IPF, and consequent risk of misdiagnosis, could have affected data collection.

## Conclusions

In summary, we developed a multidimensional PCR index/grading system and calculator that predict 3-month mortality in patients with AE-IPF. We believe that the PCR models are complimentary and have clinical and research utility. In addition, a future study should evaluate the present scoring system in a clinical setting. Finally, a large-scale multicenter study with a validation cohort is necessary in order to confirm the validity of the present results.

## Abbreviations

IPF: Idiopathic pulmonary fibrosis
AE-IPF: Acute exacerbation of idiopathic pulmonary fibrosis
ROC: Receiver operating characteristic
CRP: C-reactive protein
HRCT: High resolution computed tomography
LDH: Lactate dehydrogenase
KL-6: Krebs von den Lungen-6
SP-D: Surfactant protein D
UIP: Usual interstitial pneumonia
GAP index: Gender-age-physiology index
FVC: Forced vital capacity
DLco: Diffusing capacity of the lung for carbon monoxide
OR: Odds ratio
AUC: Area under the ROC curve
